# Unprovoked or provoked venous thromboembolism: not the prevalent criterion to decide on anticoagulation extension in clinical practice of various countries—the prospective, international, observational WHITE study

**DOI:** 10.1007/s11739-021-02765-1

**Published:** 2021-07-27

**Authors:** Gualtiero Palareti, Angelo Bignamini, Michela Cini, Young-Jun Li, Tomasz Urbanek, Juraj Madaric, Kamel Bouslama, German Y. Sokurenko, Giuseppe M. Andreozzi, Jiří Matuška, Armando Mansilha, Victor Barinov, for the WHITE study group, Jian-long Liu, Fu-xian Zhang, Tao Yang, Wei Ye, Qi Wang, Xiangchen Dai, Zhen Li, Jian Zhang, Yong-jun Li, Jin-song Wang, Ju He, Yi-qing Li, Xiao-qiang Li, Zhong Chen, Ai-min Qian, Monika Vogelová, Poliklinika Modřany, Stanislava Králová, Jana Hirmerová, Jiří Matuška, Stanislav Šárník, Marcin Gabriel, Wojciech Sydor, Filip Szymański, Tomasz Urbanek, Gabinet Lekarski, Zbigniew Krasiński, Adam Zieliński, Anna Sojda-Szarnecka, Tomasz Pelak, Wojciech Skibiński, Włodzimierz Hendiger, Tomasz Aleksiejew-Kleszczyński, Tomasz Grzela, Andrzej Szuba, Tomasz Zubilewicz, Eugeniusz Majewski, Jerzy Głowiński, Michał Molski, Krzysztof J. Filipiak, Marcin Kucharzewski, Rita Augusto, João Vaconcelos, Isabel Cássio, Ana Garcia, António Simões, Yuriy Chervyakov, Larisa Lyudkova, Victor Barinov, Ilya Schastlivtsev, Oleg Guzhkov, Sergey Katorkin, Tigran Lazaryan, Igor Prostov, Oleg Skorobogatov, Elena Burleva, Anton Scarubsky, Alexey Petrikov, Georgy Smirnov, Alexey Fokin, Igor Sonkin, Sergey Bushnin, Elena Murzina, Vladimir Zelinsky, Alexandr Bogdan, Roman Bredikhin, Ovsep Mandzhikian, Anton Isaev, Martina Padúchová, Andrej Džupina, Viera Štvrtinová, Melinda Malá, Juraj Maďarič, Ľubomíra Javorčíková, Dasa Kmecová, Meya Abdelkefi, Kamel Bouslama, Raja Elamri, Fatma Boussema, Chedia Kechrid

**Affiliations:** 1 Arianna Anticoagulazione Foundation, Via Paolo Fabbri 1/3, 401138 Bologna, Italy; 2 School of Specialization in Hospital Pharmacy, University of Milan, Milan, Italy; 3 Peking Union Medical College, Chinese Academy of Medical Sciences, Beijing, China; 4 Medical University of Silesia, Katowice, Poland; 5 Clinic of Angiology, Comenius University and National Institute of Cardiovascular Diseases, Bratislava, Slovakia; 6 Faculty of Medicine of Tunis, Tunis, Tunisia; 7 North-West Mechnikov State Medical University, St. Petersburg, Russia; 8 Angiology Unit, University of Padua, Padua, Italy; 9 Clinical Trial Centre, Hodonin, Czech Republic; 10 Faculty of Medicine, University of Porto, Porto, Portugal; 11 Central State Medical Academy of the Office of the President of the Russian Federation, Clinical hospital N. 1 “Volynskaya”, Moscow, Russia

**Keywords:** Venous thromboembolism, Anticoagulation, Anticoagulants, Antithrombotics, Sulodexide, Aspirin

## Abstract

The decision on treatment after a first venous thromboembolism (VTE) to prevent recurrences may be influenced by many factors. The prospective, observational, WHITE study aimed to analyze how this issue was tackled in every-day clinical practice in various countries, which have sensibly different socio-economic conditions and healthcare systems. Doctors active in 79 Internal or Vascular clinical centers in 7 countries (China, Czechia, Poland, Portugal, Russia, Slovakia, and Tunisia) enrolled VTE patients after the maintenance treatment phase. The present report analyzed information, collected in the central database, regarding the baseline characteristics, index events, type and duration of anticoagulant therapy and decision on post-maintenance treatment. From April 2018 to December 2020, 1240 patients were enrolled, 58% with an unprovoked index event. Direct oral anticoagulants (DOACs) were used in > 85% of all cases in China, Poland, Portugal, Russia and Czechia, in 52% in Slovakia and in no patient in Tunisia. The maintenance anticoagulation lasted in average approximately 6 months. Altogether, anticoagulation was stopped in 20%, extended in about 50%, regardless of whether the event was unprovoked or provoked and shifted to antithrombotics (mainly sulodexide or aspirin) in the remaining patients. In conclusion, some differences in VTE patient management were found between countries. The provoked/unprovoked nature of the index event, instead, was not the prevalent criterion to drive the decision on extension of anticoagulation, without large variations between countries. DOACs were the most widely used anticoagulant drugs, whereas > 25% of patients received antithrombotic drugs instead of anticoagulants as extended treatment.

**Supplementary Information:**

The online version contains supplementary material available at 10.1007/s11739-021-02765-1.

## Introduction

The guidelines [1] recommend initial treatment of deep vein thrombosis (DVT) of the lower limbs and/or pulmonary embolism (PE) with a parenteral direct-acting anticoagulant or direct oral anticoagulant (DOAC), followed by a period of anticoagulation therapy with a vitamin K antagonist (VKA) or, preferably, a DOAC, or low molecular weight heparin (LMWH), particularly in patients with cancer-associated thrombosis. Anticoagulation therapy is essential for at least 3–6 months in all cases [2], whereas a longer treatment is intended to decrease the risk of recurrences. The incidence of venous thromboembolism (VTE) recurrence is very low when an event is provoked by surgery or another specific strong and removable risk factor. In these cases, a three- to six-month treatment (defined as “maintenance” treatment period) is considered sufficient. Conversely, the risk of recurrence is reported very high in subjects with permanent and strong risk factors, such as cancer, inflammatory diseases, serious acquired or inherited thrombophilic alterations, repeated VTE events, or when the first event was life-threatening. In these cases, an indefinite anticoagulant treatment is recommended. A significant number of VTE patients, however, have events without any apparent risk factors (unprovoked), or associated with weak risk factors. In these patients, an anticoagulant treatment limited to the maintenance phase may not be sufficient and international guidelines suggest an extended (indefinite, i.e., without a predetermined stop date) anticoagulation, provided that the risk of bleeding associated with anticoagulation is not high [1].

In line with international guidelines, at the end of the maintenance period, all patients with an acute VTE event, should be evaluated for their risk of recurrence and of bleeding, and the attending physician must decide what to do next. The possible options are (a) interrupt any specific pharmacological treatment; (b) continue with extended anticoagulation using the same or another anticoagulant drug, at standard or reduced dosage; or (c) replace the anticoagulant in use with an alternative antithrombotic drug. However, the individual patient’s risks are not always easy to be estimated. A number of additional factors may also influence the clinical decision, including subject’s preferences, concurrent diseases and treatments, healthcare system support, and availability of potentially effective treatments alternative to anticoagulation. In addition, based on the local healthcare system, not all regimens can be protracted indefinitely in all cases. Little is known as to which proportion of subjects is assigned to these alternatives and whether this proportion is equivalent across different healthcare systems. After the decision is taken, a number of validated data are available in the literature regarding the outcomes in subjects assigned to extended anticoagulation for up to 1 year [3–9], whereas little if any information is available for subjects receiving extended anticoagulation for more than one year, or assigned to alternative long-term treatment, or who stopped the treatment. Furthermore, little is known about the actual practice concerning this issue in countries of different geographic areas, differing for socio-economic conditions and healthcare systems.

The international WHITE study was intended to analyze data on these issues collected from the every-day clinical practice in various countries which have sensibly different socio-economic conditions and healthcare systems.

## Materials and methods

### Study design, participants, and study population

WHITE was a multicenter, multinational, observational, non-interventional, investigators-initiated, no-profit, prospective study (ClinicalTrials.gov Identifier: NCT04646993), dedicated to evaluating the decisions taken by clinicians at the end of the maintenance treatment of subjects with a first-ever event of DVT of the lower limbs and/or pulmonary embolism. The study was an independent research initiative promoted by the “Arianna Anticoagulazione” Foundation (Bologna, Italy), and managed in collaboration with a Core Team of vascular-experts professionals (the detailed list of the study boards and their compositions is shown in Appendix 1). One Country Coordinator for each active country, appointed by the Foundation, invited local clinical vascular centers to participate in the study, and collaborated with local contract research organizations (CRO) to obtain authorization to the study from the national Health Authority Boards and the local Ethics Committees. The study was carried out and reported according to the “Strengthening the Reporting of Observational Studies in Epidemiology (STROBE)” guidelines for observational studies [10].

The study aimed at enrolling patients during the maintenance treatment after diagnosis of a first DVT and/or PE event. Patients of any ethnicity, male and female, aged ≥ 18 years, were eligible for the study if treated with anticoagulant therapy for 3–12 months following a documented first-ever event of provoked or unprovoked DVT of the lower limbs and/or of PE. Subjects provided a written informed consent before inclusion in the study. Excluded from the study were subjects < 18 years old, with thrombosis in other sites, unable or unwilling to give written informed consent (complete list of inclusion/exclusion criteria in the Protocol, available on request). Each subject had the right to refuse continuing the study at any moment and without justifications.

The primary objective of the study was to assess the clinicians’ decisions on the modality to manage the secondary prevention in patients after a first VTE episode, at the end of the maintenance period of anticoagulation considered standard, and the reasons guiding the physician’s decision. Secondary objective was the recording of thromboembolic or bleeding complications, and of deaths occurring during follow-up. The study encompassed two sections: (a) a transversal section, in which the primary objectives were evaluated; and (b) a longitudinal section, in which the secondary objectives were evaluated. The type, dose and duration of patients’ treatment before and after inclusion in the Study were left at the attending physician’s discretion.

### Data collection and management

All participants in the WHITE study recorded the information collected in a structured case report form (CRF;) onto a secure, web-based central electronic database designed and managed by Officinebit (Switzerland) and were controlled at the study coordinating center (Arianna Anticoagulazione Foundation) by a dedicated study-monitor (C.M.) who—when necessary—sent data queries to participating sites to ensure completeness and accuracy. All patients were assigned a unique identifier, and personally identifiable data were removed at the participant’s side to ensure anonymity.

The scope of the present analysis is limited to the transversal section of the study, including the baseline patient conditions, the index VTE events with associated risk factors, the maintenance treatment of the events, and the clinicians’ decisions on the management of patients regarding the secondary VTE prevention. The site of index VTE events was classified in (a) DVT, when thrombosis involved proximal veins (extending or not to calf deep veins) without diagnosed PE; (b) IDDVT, when thrombosis was limited to calf (distal) deep veins; (c) PE (± DVT), when an objectively documented PE occurred in the absence or in presence of a DVT (proximal or distal). The available data regarding the objective conditions of the affected lower limb with assessment of post-thrombotic syndrome and the longitudinal section of the study, including the events recorded during follow-up in patients after they received the management decision will be reported subsequently.

### Statistical analysis

Data have been analyzed with SPSS version 24 integrated for specific items with R version 3.6.1. All variables have been summarized with the usual descriptive techniques. Demographic and clinical variables were compared, when needed, with the Chi-square or Fisher’s test for nominal variables, and ANOVA, integrated where appropriate with the post-hoc pairwise Tukey HSD test, for the continuous variables. Only when absolutely needed, we used the non-parametric approach (Mann–Whitney and Kruskal–Wallis tests). The proportion of choices made by the attending physician was reported as proportion with confidence interval. The primary analysis tested by goodness-of-fit whether the choices were random or not. The impact of demographic and prognostic factors—including the country—on the choice was tested by chi-square and, where appropriate, logistic regression analysis.

### Funding

The “Arianna Anticoagulazione” Foundation (Bologna Italy) promoted the WHITE study. Public and private institutions, companies and individuals interested in the issue of anticoagulant or antithrombotic treatments (manufacturers of drugs or other goods and services) were asked by the Executive Committee of the Foundation to help fund the promoted studies via unrestricted grants without any right to access the database. Members of the Foundation’s Executive Committee do not receive any payment or fee for their work with the Foundation. The Foundation has received an unrestricted research grant from Alfasigma (Bologna, Italy), specifically dedicated to the realization of this study.

### Availability and cost of VTE treatments in the countries involved in the study

The specificities of the individual healthcare systems were considered factors potentially able to influence the monitored outcomes. A pre-study survey, performed with the participation of the National Coordinators, singled out similarities but also important differences. VKA anticoagulants were available in all countries and free of charge except in Tunisia. LMWH were equally available in all countries and free of charge except in Poland, Slovakia, and Tunisia, where they were available at an average daily cost of € 1, € 5, and € 8, respectively. The status of the direct oral anticoagulants (DOACs) was highly variable. No DOAC was being marketed in Tunisia, whereas all DOACs were available in Czechia and, except edoxaban, in China and Slovakia. Rivaroxaban was available in all countries; in Poland, also dabigatran was available. DOACs were free of charge in Czechia and almost free in Slovakia. The cost of one month of DOAC treatment at standard dose was of € 25–30 and € 35 for rivaroxaban and dabigatran, respectively, in Poland, and of € 120 in Russia for rivaroxaban. In China, the daily treatment cost with DOACs ranged from 10 to 20 euro. Low-dose ASA (Cardioaspirin^®^) was available in all countries and free of charge except in Tunisia (€ 1 per month) and Poland (€ 2 per month). Sulodexide was available in all countries but in none was it free of charge. Follow-up visits for VTE patients were recommended by all the National Healthcare Systems except in China and were free of charge in Czechia, Poland, and Slovakia.

## Results

**Fig. 1:**
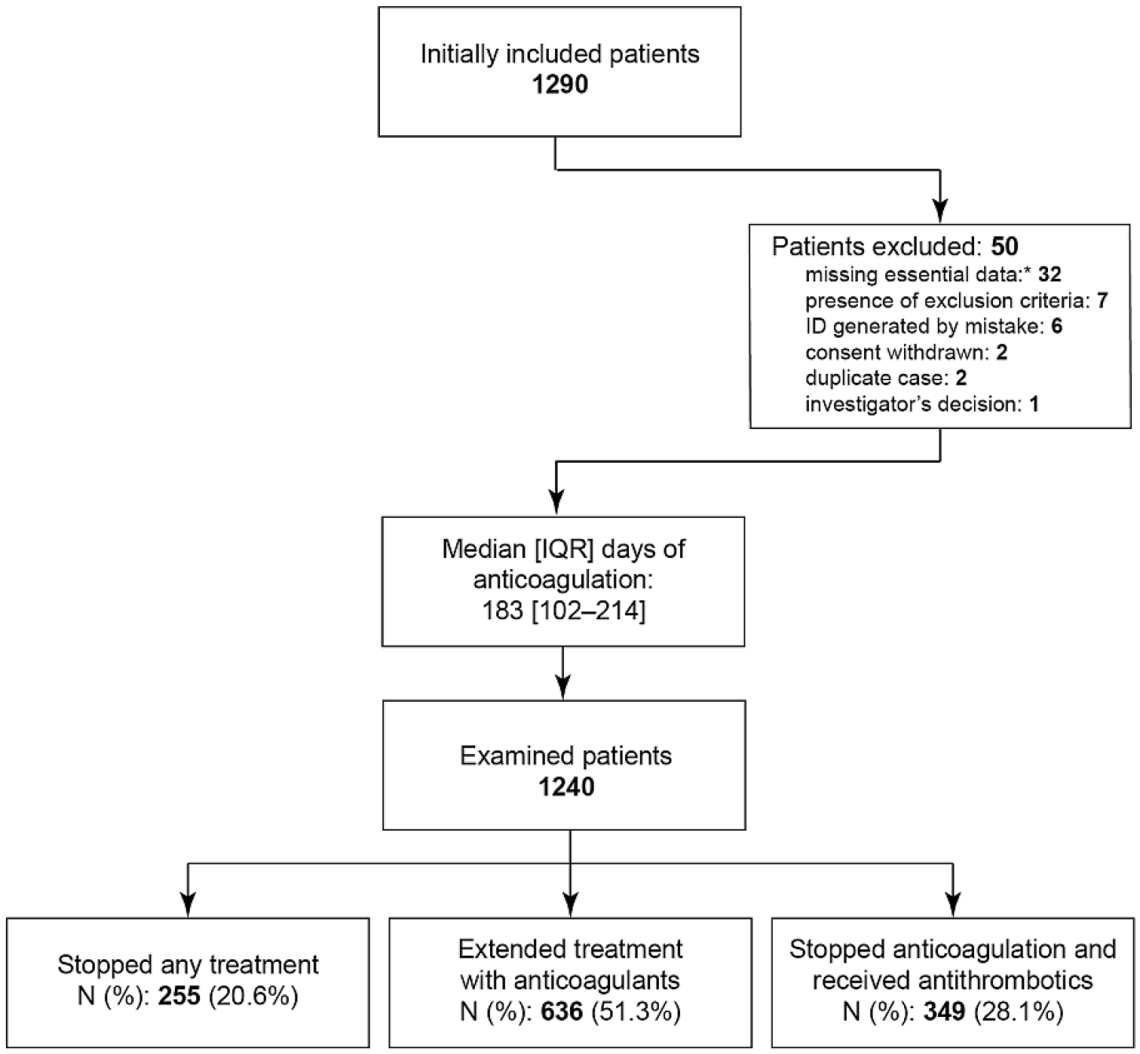
Flow chart of investigated patients

The enrollment of patients started on April 2018. Recruitment was severely delayed by slow bureaucratic approval procedures (particularly in some countries) and the effects of SARS-CoV-2 pandemics, that substantially limited the capacity of recruitment. The Foundation decided to stop inclusion of new patients on December 15, 2020, when 1240 valid patients (Fig. 1) had been recruited by 79 active clinical sites (the complete list of active clinical centers is reported below), in 7 countries (Table 1).

**Table 1:** Active clinical sites and enrolled patients in each country; demographic information, clinical characteristics of included patients and type of index events

Country	Active sites, *n*	Patients enrolled, *N*. (%)	Females, *N* (%w)	Age, years mean ± SD (*N*)	BMI mean ± SD (*N*)	Hypert., *N* (%w)	Diabetes, *N* (%w)	IHD, *N* (%w)	CVD, *N* (%w)	Kidney failure, *N* (%w)	Smoking, *N* (%w)	Proximal DVT (± distal DVT) *n*. (%w)	Isolated distal DVT *n*. (%w)	PE (± DVT) *n*. (%w)
China	15	317 (25.6%)	162 (51.1%)	55.6 ± 15.1 (*N* = 317)	24.97 ± 3.36 (*N* = 312)	90 (28.4%)	40 (12.6%)	22 (6.9%)	14 (4.4%)	1 (0.3%)	57** (18.1%)	148 (46.7%)	134 (42.3%)	35 (11.0%)
Czechia	5	70 (5.6%)	40 (57.1%)	54.2 ± 15.7 (*N* = 70)	27.93 ± 5.14 (*N* = 67)	27 (38.6%)	8 (11.4%)	3 (4.3%)	1 (1.4%)	0 (0.0%)	16 (22.9%)	20 (28.6%)	29 (41.4%)	21 (30.0%)
Poland	20	133 (10.7%)	59 (44.4%)	56.5 ± 16.7 (*N* = 133)	28.38 ± 5.12 (*N* = 114)	57 (42.9%)	13 (9.8%)	15 (11.3%)	1 (0.8%)	7 (5.3%)	17* (12.9%)	47 (35.3%)	55 (41.3%)	31 (23.3%)
Portugal	5	41 (3.3%)	22 (55.0%)	58.8 ± 17.9 (N = 41)	28.32 ± 4.39 (*N* = 38)	18 (43.9%)	4 (9.8%)	2 (4.9%)	1 (2.4%)	1 (2.4%)	6 (14.6%)	24 (58.5%)	13 (31.7%)	4 (9.8%)
Russia	22	501 (40.4%)	253 (50.6%)	57.4 ± 14.9 (*N* = 501)	28.17 ± 5.37 (*N* = 499)	229 (45.7%)	48 (9.6%)	84 (16.8%)	16 (3.2%)	4 (0.8%)	111^§^ (24.0%)	328 (65.5%)	132 (26.3%)	41 (8.2%)
Slovakia	7	98 (7.9%)	47 (48.0%)	56.7 ± 15.2 (*N* = 98)	28.46 ± 4.00 (*N* = 97)	42 (42.9%)	9 (9.2%)	6 (6.1%)	1 (1.0%)	2 (2.0%)	11 (11.2%)	49 (50.0%)	42 (42.9%)	7 (7.1%)
Tunisia	5	80 (8.5%)	44 (55.7%)	56.4 ± 17.4 (*N* = 80)	29.96 ± 5.37 (*N* = 76)	22 (27.5%)	15 (18.8%)	4 (5.0%)	1 (1.3%)	3 (3.8%)	17* (21.5%)	36 (45.0%)	25 (31.3%)	19 (23.8%)
Total	79	1240 (100.0%)	627 (50.7%)	56.6 ± 15.5 (*N* = 1240)	27.49 ± 4.99 (*N* = 1203)	485 (39.1%)	137 (11.0%)	136 (11.0%)	35 (2.8%)	18 (1.5%)	235 (19.6%)	652 (52.6%)	430 (34.7%)	158 (12.7%)
Statistics *P*			0.582^b^	0.512^a^	< 0.001^a^	< 0.001^b^	0.288^b^	< 0.001^b^	0.264^b^	0.001^b^	0.016^b^	< 0.001^b^		

^a^ANOVA

^b^Chi square

*1 case N/A;

**2 cases N/A:

^§^39 cases N/A

(%w) denotes percent within country, i.e., proportion of the total number of subjects recruited in that country with the indicated characteristic

BMI denotes body mass index in kg/m^2^; Hypert. denotes hypertension; IHD denotes ischemic heart disease; CVD denotes cerebrovascular disease

### Baseline characteristics and index events

Caucasian subjects were the most prevalent (69%). Age and sex distribution did not differ among countries, whereas BMI was significantly lower in Chinese subjects versus all the others (Table 1). The prevalence of hypertension was lowest in Tunisia and China (about 28% in both countries), and highest in Russian patients (45.7%), who also reported the highest prevalence of ischemic heart disease (16.8%) and of smokers (24%). No differences were detected among countries for the presence of diabetes or cerebrovascular diseases. Only 18 patients reported kidney failure, almost one-third of whom (5 cases) from Poland.

The distribution of the index event (Table 1) was significantly different by country (*P* < 0.001), essentially because the rate of the reported PE in the patients included into the study in Czechia was 1.6 to 3 times greater than in all other countries (*P* = 0.005). Among the patients with DVT, the most affected limb was the left (59% vs. 45% of cases with right limb affected; *P* < 0.001).

**Fig. 2:**
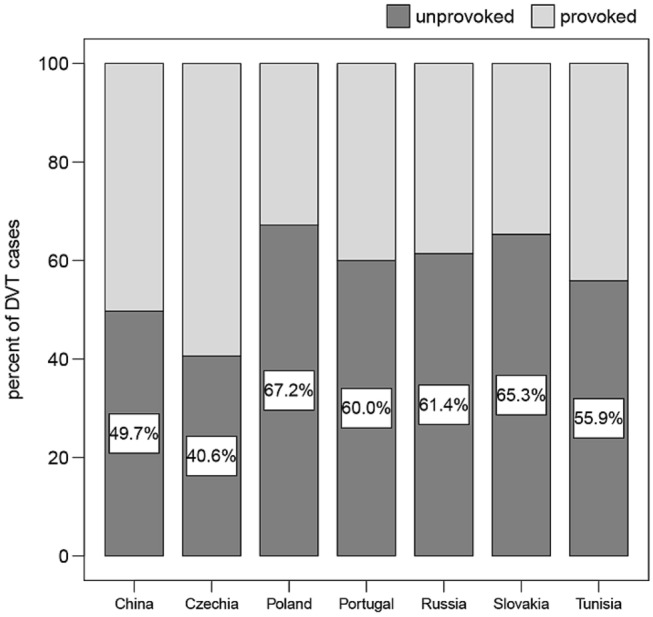
Distribution by country of the index events as provoked or unprovoked

The VTE events were classified unprovoked in 696 cases (58%) and provoked in the balance. The classification differed significantly across countries (*P* < 0.001), with the proportion of unprovoked DVTs significantly below average in China and Czechia (Fig. 2). The distribution of risk factors for thrombosis agreed with the distribution as provoked or unprovoked (Supplemental Table S-1), whereas no major differences were seen by index event (Supplemental Table S-2).

### Treatment during maintenance phase

The classes of anticoagulant drugs used during the maintenance treatment phase differed between countries. The distribution of treatments privileged the direct oral anticoagulants (DOACs), which were used in 86–94% of cases in China, Poland, Portugal, Russia, and Czechia, 52% in Slovakia and were not used in Tunisia, where VKAs were instead used in 86% of subjects. There was no difference of treatment by index event (Supplemental Table S-3), nor by type of event, although the IDDVT represented a significantly greater fraction of the provoked events (Supplemental Table S-4).

**Fig. 3:**
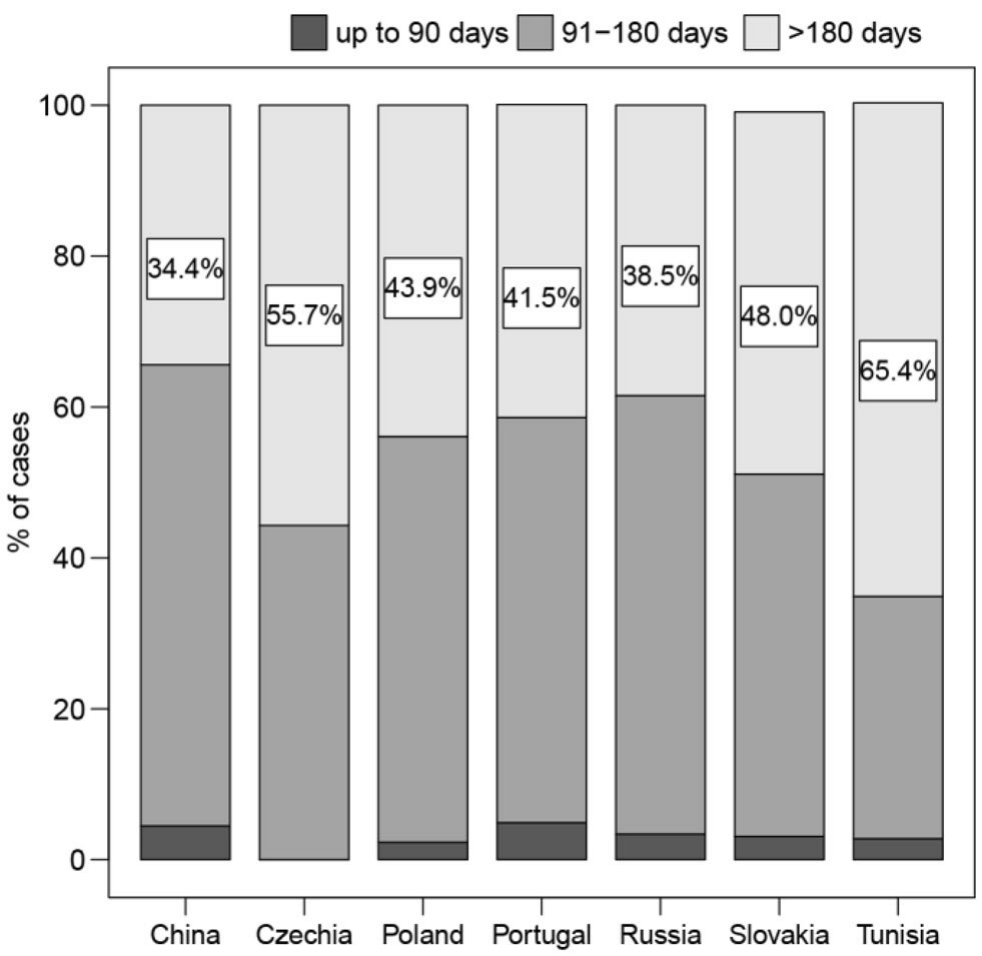
Distribution by country of maintenance anticoagulation duration

In average, the maintenance anticoagulation had lasted approximately 6 months at the time of the decision; however, it was the shortest in China (mean ± SD: 164 ± 83 days) and the longest in Tunisia (205 ± 68) and Czechia (196 ± 82). The anticoagulation duration in case of unprovoked events was in average slightly longer than in provoked events, but the difference was statistically significant only in Czechia (*P* = 0.006) and Slovakia (*P* = 0.007). Only very few patients (none in Czechia) received a maintenance anticoagulation for < 90 days, while a treatment for > 180 days was given to a proportion of patients ranging from 34.4% (China) to 65.4% (Tunisia; Fig. 3).

### Decision about extension of treatment

In about 20% of patients, the attending physician decided to stop the anticoagulant treatment (Table 2); an extension of anticoagulation (whatever the drug used) was decided in more than half the patients (51.3%), whereas in 28.1% of them, an antithrombotic drug (sulodexide or antiplatelets) was prescribed. The distribution of the decisions clearly differed from the uniform distribution (*P* < 0.001, goodness-of-fit test), and differed significantly among countries. Discontinuation without replacement was decided in 27–40% of cases in Portugal, Tunisia, and Czechia, vs. 3% of cases in Slovakia. An extended anticoagulation was decided in 51–68% of cases in Poland, Russia, China, and Portugal, vs. 25–26% in Czechia and Tunisia. Continuation with other antithrombotics was decided in 38–60% of cases in Poland, Tunisia, and Slovakia vs. 5% in Portugal. A DOAC was used in 85% of cases who extended anticoagulation; when continuing with the same anticoagulant, the dose was reduced in 17% of cases. Switching from one to another DOAC was reported in only 19 subjects. Overall, 351 subjects switched from the maintenance anticoagulation to prophylaxis with antithrombotic agents: sulodexide in 275 cases (57% with the dose of 500 LSU/day and the balance with 1000 LSU/day), ASA in 72 (75–175 mg/day), clopidogrel in 6, and other treatments in 8.

**Table 2:** Distribution of clinical decisions for the treatment of patients beyond the maintenance phase

	Anticoagulant treatment was stopped, *N* (%)	Anticoagulant treatment was extended, *N* (%)	Treatment continued with antithrombotic agents, *N* (%)
China *N* = 317	76 (24.0%)	190 (59.9%)	51 (16.1%)
Czechia *N* = 70	28 (40.0%)	18 (25.7%)	24 (34.3%)
Poland *N* = 133	15 (11.3%)	68 (51.1%)	50 (37.6%)
Portugal *N* = 41	11 (26.8%)	28 (68.3%)	2 (4.9%)
Russia *N* = 501	95 (19.0%)	275 (54.9%)	131 (26.1%)
Slovakia *N* = 98	3 (3.1%)	36 (36.7%)	59 (60.2%)
Tunisia *N* = 80	27 (33.8%)	21 (26.3%)	32 (40.0%)
Total *N* = 1240	255 (20.6%)	636 (51.3%)	349 (28.1%)

As shown in Table 3, anticoagulation was stopped in 15.4% and 28.9% and continued in 51.7% and 49.3% of patients who had unprovoked or provoked events, respectively. Anticoagulation was stopped in a minority of cases (about 13%) and extended in more than half of patients when the index events were proximal with or without distal DVT, or PE (with or without DVT). Anticoagulation was stopped in one-third and extended in a similar proportion of IDDVT cases. The prescription of antithrombotics was higher when the event was unprovoked (32.9%) vs provoked (21.8%, *P* < 0.001), and similar (about one-fourth of patients) in all types of events. The availability of recent ultrasound close to the decision favored the continuation with other non-anticoagulant antithrombotics rather than with anticoagulation; the same occurred among the unprovoked events, while among the provoked events it had no influence on the continuation with anticoagulation, whereas the absence of recent ultrasound favored stopping treatment (Supplemental Table S-5).

**Table 3:** Decisions about the treatment after the maintenance phase

Decision	Event	Unprovoked,* *N* = 696	Provoked,* *N* = 509	*P* (chi-square)	Proximal ± distal DVT, *N* = 652	PE with DVT, *N* = 123	Isolated distal DVT, *N* = 430	PE without DVT, *N* = 35	*P* (chi-square)	All cases**, *N* = 1240
Anticoagulant treatment was stopped, *N* (%)		107 (15.4%)	147 (28.9%)	< 0.001	86 (13.2%)	20 (16.3%)	148 (34.4%)	1 (2.9%)	< 0.001	
Anticoagulation was extended, N (%)		360 (51.7%)	251 (49.3%)	0.408	379 (58.1%)	73 (59.3%)	159 (37.0%)	25 (71.4%)	< 0.001	
Antithrombotic drugs were prescribed, *N* (%)		229 (32.9%)	111 (21.8%)	< 0.001	187 (28.7%)	30 (24.4%)	123 (28.6%)	9 (25.7%)	0.778	
Anticoagulant treatment was stopped, *N* (%)	Cases	107	147							255
	proximal ± distal DVT	41 (38.3%)	45 (30.6%)	0.378						86 (33.7%)
	isolated distal DVT	57 (53.3%)	91 (61.9%)							148 (58.0%)
	DVT + PE	9 (8.4%)	11 (7.5%)							20 (7.8%)
	PE without DVT	–	–							1 (0.4%)
Anticoagulation was extended, N (%)	Cases	360	251							636
	proximal ± distal DVT	225 (62.5%)	154 (61.4%)	0.951						379 (59.6%)
	isolated distal DVT	93 (25.8%)	66 (26.3%)							159 (25.0%)
	DVT + PE	42 (11.7%)	31 (12.4%)							73 (11.5%)
	PE without DVT	–	–							25 (3.9%)
Antithrombotic drugs were prescribed, *N* (%)	Cases	229	111							349
	proximal ± distal DVT	130 (56.8%)	57 (51.4%)	0.191						187 (53.6%)
	Isolated distal DVT	76 (33.2%)	47 (42.3%)							123 (35.2%)
	DVT + PE	23 (10.0%)	7 (6.3%)							30 (8.6%)
	PE without DVT	–	–							9 (2.6%)

*In 35 subjects with isolated PE, the classification of the index event as provoked or unprovoked was not collected

**Including the 35 events of isolated PE

We examined whether the main demographic and prognostic factors might have influenced the decision taken. Sex, age and presence of cardiovascular risk factors did not significantly affect the choice of the regimen. The odds to continue with anticoagulation rather than stopping were higher among subjects with PE vs. those with only DVT [2.71 times (1.59–4.61; *P* < 0.001)] and in those with concomitant diseases [1.49 (1.07–2.06; *P* = 0.018)]. Limiting the analysis to the subjects with DVT, the odds to continue with anticoagulation rather than stopping was 1.88 [1.37–2.58; *P* = 0.001] among subjects with unprovoked DVT vs. those with provoked DVT and was 5.25 [3.32–8.31; *P* < 0.001] in subjects with vs. those without PTS signs. The odds to continue with other antithrombotics rather than stopping were higher in subjects with concomitant diseases [1.62 times (1.10–2.36; *P* < 0.001)], in those with unprovoked DVT [2.62 (1.83–3.75; *P* < 0.001)] and in those with signs of PTS [3.04 (1.78; 5.22; *P* < 0.001)]. The complete results of the logistic regression analysis are reported in Supplemental Table S-6. We also collected information on the weight of other motivations as potential predictors of decision (Supplemental Table S-7), which will not be discussed here.

## Discussion

This prospective study examined 1240 patients who had suffered from a first DVT and/or PE event and were enrolled during maintenance anticoagulation treatment in 79 clinical centers active in seven countries which differed by geographic area, socio-economic conditions, and health care systems. The main aims of the study were to investigate how the patients were managed in these countries, which anticoagulants and for how long were used for the maintenance treatment phase and which decision was taken about treatment after that phase. As expected, many differences were found among the countries and some similarities. While age and sex distribution did not differ in patients of the involved countries, the prevalence of hypertension and IHD was the highest in Russia, the country which also had the highest prevalence of smokers (24%). The distribution of the index event reported in the study was significantly different by country. The most prevalent index event was proximal DVT (with or without involvement of distal veins), followed by isolated distal DVT; less frequent were cases of PE with or without DVT. DVT events involved 1.3 times more frequently the left rather than the right limb.

In all countries except Tunisia (where these drugs were not available), DOACs were the type of anticoagulant drug more often adopted for treatment during maintenance phase (Table 2); their use ranged between 52% (Slovakia) and 94.3% of patients (Czechia). Overall, DOACs were adopted in 84% of all patients (excluding those from Tunisia), a prevalence quite similar to that (79.5%) recorded in a cohort of Italian VTE patients recently described [11]. These data show how widespread is the current DOAC use for VTE treatment in many countries (when available) and confirm the large preference for DOACs by treating physicians and patients as well.

In line with the international guideline [1], very few patients received a maintenance anticoagulant treatment for less than the recommended 90 days. The prevalent portion of patients received anticoagulant treatment for 3–6 months; however, a consistent portion of all patients (about 40%) were treated for > 180 days before being considered for possible extension of anticoagulation. The prevalence of a long-lasting maintenance treatment was particularly evident in Tunisia (65.4% of patients), where anticoagulation was performed with VKAs, and in Czechia (55.7%), where DOACs were free. The mean duration of maintenance anticoagulation was not different between patients with unprovoked or provoked events in all the countries, with exception of Czechia and Slovakia, where unprovoked VTE patients were treated for a significantly longer period.

Not many studies have assessed the treatment of VTE patients in real-world populations. Among Italian VTE patients, prevalently managed by vascular doctors, an anticoagulation for < 3 months was recorded in more patients (9%) than found in the present study; conversely, quite similar (36.8%) was the prevalence of patients who were treated for > 180 days [12]. Completely different results were reported in a Canadian study [13], in which only 73% of patients with proven VTE were treated with anticoagulation (with VKAs) and for a much shorter period (median 61 days). These data, as well as those reported in the present study, depict how big are the differences among countries in the routine management of VTE patients in real-world settings.

The physicians’ decision about what to do after the maintenance anticoagulation period was one of the main issues addressed in the present study. Anticoagulant treatment was stopped in only one-fifth of all included patients, whereas anticoagulant therapy was extended in about half of them, and an indication for continuing treatment using various antithrombotic agents was given to the remaining patients. Though with important differences among countries, the therapeutic decisions did not seem to be predominantly influenced by the unprovoked or provoked nature of the index event, as shown by the fact that anticoagulation was extended in the same proportion of the two types of patients (51.7% vs. 49.3%, respectively). This appears to be in contrast with what suggested by experts [2] and international guidelines [1] which have recommended at least 3 months of anticoagulation for all VTE patients and to consider extension of treatment in those with unprovoked events who have a non-high risk of bleeding. However, this result, fully consistent with what Italian vascular doctors decided in a cohort of VTE patients [12], indicates that many physicians in the everyday clinical practice of different countries prefer not to comply with a pretended obligation dictated by the classification as unprovoked/provoked event, but rather to try and assess the multifactorial individual recurrence risk. Indeed, at the multivariable analysis, the provoked/unprovoked nature of the event along with its site (PE or not), the presence of PTS signs and the presence of concomitant diseases, significantly affected the probability to continue with anticoagulation or antithrombotic treatment, rather than stopping. In contrast with international guidelines [1] and experts [2, 14] which indicate that male patients are at higher risk of recurrences than females and therefore that sex can be used to stratify patients for their risk and decide the extension of anticoagulation, in the present study, sex was not a factor determining the therapeutic decision, as well as age and presence of risk factors for cardiovascular diseases.

Another relevant result of this study is that many participant physicians seemed to be worried about completely stopping any treatment after the maintenance phase and limited this decision only to patients considered at low risk of recurrence, especially to those with IDDVT (31% of them stopped any treatment). An extended anticoagulant treatment was prescribed preferably to patients estimated at very high risk of recurrence; whereas treatment with antithrombotic agents, mainly sulodexide or aspirin, was suggested to many patients, likely to maintain a kind of protection against recurrences though using drugs at lower risk of bleeding, and probably also at lower cost.

### Limitations

Our study has important limitations. Patients were included from clinical centers active in countries which presented big differences, for many important aspects, differences that were impossible to adjust. Furthermore, the number of involved centers and of patients included in each country do not allow to draw general conclusions on the clinical practice adopted in each country. Finally, patient information was collected in a prospective observational registry in which all the therapeutic decisions were left to the attending physicians. For these reasons, the interpretation of our findings requires caution. We believe, however, that in the present “global” world, an effort to assess which is a prevalent approach to VTE patient management in the real-world setting of different countries is of value. It is worth promoting a comparison of everyday therapeutic procedures adopted in different countries.

In conclusion, this study provided information prospectively collected on the management of patients with recent VTE, included from clinical centers active in seven countries that are different for many important aspects. As expected, many differences in VTE patient management were found among the countries and also some similarities. DOACs were the most widely used anticoagulant drugs that most patients received for maintenance anticoagulation up to 180 days. After maintenance period, any treatment was stopped in 20% of patients, whereas anticoagulant drugs were continued in half of patients, regardless of the classification of the index event as unprovoked or provoked; antithrombotic drugs (especially sulodexide and aspirin) were prescribed to the remaining patients.

### Electronic supplementary material

Below is the link to the electronic supplementary material.

Supplementary file1 (DOCX 28 kb)

## List and composition of the study boards

### Core team

**Table:**

Core Team Member	Prof. Jiří Matuška Clinical Trial Centre, Hodonin, Czechia e-mail: jiri.matuska@matmed.cz
Core Team Member	Prof. German Y. Sokurenko North-West Mechnikov State Medical University, St. Petersburg, Russia e-mail: german_sokurenko@mail.ru
Core Team Member and DMSB Member	Prof. Gualtiero Palareti University of Bologna (ret.), Italy e-mail: gualtiero.palareti@unibo.it
Core Team Member and DMSB Member	Prof. Giuseppe Maria Andreozzi Past Director, Angiology Unit, University of Padua, Italy e-mail: gm.andreozzi@gmail.com
Core Team Member and DMSB Member	Prof. Angelo A. Bignamini University of Milan, Italy e-mail: angelo@aabignamini.it

### Responsible parties

**Table:**

Promoter	Prof. Gualtiero Palareti President, Fondazione Arianna Anticoagulazione	info@fondazionearianna.org
International Study Coordinator and National Study Coordinator, Czechia	Dr. Jiří Matuška Clinical Trial Centre, Hodonin, Czechia	jiri.matuska@matmed.cz
National Study Coordinator, Russia	Prof. Victor Barinov Department of Surgery of Central State Medical Academy of the President Administration of Russian Federation, Clinical hospital N. 1 “Volynskaya”, Moscow, Russia	vicbarin@mail.ru
National Study Coordinator, Tunisia	Prof. Kamel Bouslama Faculty of Medicine of Tunis, Tunisia	kamel.bouslama387@gmail.com
National Study Coordinator, China	Prof. Young-Jun Li Peking Union Medical College, Chinese Academy of Medical Sciences, Beijing, China	liyongjun4679@bjhmoh.cn
National Study Coordinator, Slovakia	Assoc. Prof. Juraj Madaric National Institute of Cardiovascular Diseases, Bratislava, Slovakia	madaricjuraj@gmail.com
National Study Coordinator, Portugal	Prof. Armando Mansilha University of Porto, Portugal	vascular.mansilha@gmail.com
National Study Coordinator, Poland	Prof. Tomasz Urbanek Medical University of Silesia, Katowice, Poland	urbanek.tom@interia.pl
Study Statistician	Prof. Angelo A. Bignamini Dept. of Pharmaceutical Sciences University of Milan (Italy)	angelo@aabignamini.it
Study Monitor	Dr. Michela Cini Arianna Anticoagulazione Foundation Bologna, Italy	m.cini@fondazionearianna.org
Database Manager	Dr. Ilaria Caldirola OFFICINEBIT.CH sagl Chiasso, Switzerland	ilaria@officinebit.ch

CRO used in the countries:

- Medart, Warsaw, Poland
- Datamedica, Amadora, Portugal
- Value Outcomes s.r.o. Praha, Czechia
- Poseidon CRO, Ariana, Tunisia
- JD Software, Bratislava, Slovakia

No professional CROs were necessary in China and Russia, where the local Principal Investigators were able to reach the study authorization from the referent health regulatory authorities.
