# Supplementary material for: Unprovoked or provoked venous thromboembolism: not the prevalent criterion to decide on anticoagulation extension in clinical practice of various countries—the prospective, international, observational WHITE study
Source: Intern Emerg Med. 2021 Jul 27;17(1):71–82. doi: 10.1007/s11739-021-02765-1 (PMC8313672; doi:10.1007/s11739-021-02765-1)
Supplement: Supplementary file 1 — Supplementary file1 (DOCX 28 kb) [file 11739_2021_2765_MOESM1_ESM.docx]

Supplemental table S-1. Distribution of risk factors for thrombosis by classification of the index event

| risk factor | unprovoked* | provoked* | P (chi square) |
| --- | --- | --- | --- |
| surgery, N=195 | 39 (5.6%) | 156 (30.6%) | <0.001 |
| severe trauma, N=127 | 12 (1.7%) | 114 (22.4%) | <0.001 |
| cancer, N=85 | 22 (3.2%) | 61 (12.0%) | <0.001 |
| paraplegia, N=12 | 1 (0.1%) | 11 (2.2%) | <0.001 |
| limb immobilization, N=91 | 8 (1.1%) | 80 (15.7%) | <0.001 |
| central venous catheter, N=8 | 1 (0.1%) | 7 (1.4%) | 0.009 |
| chronic inflammatory dis, N=28 | 10 (1.4%) | 16 (3.1%) | 0.044 |
| bedrest >4 dd, N=144 | 18 (2.6%) | 121 (23.8%) | <0.001 |
| pregnancy, N=21 | 8 (2.3%) | 13 (4.5%) | 0.127 |
| puerperium, N=8 | 4 (1.2%) | 3 (1.0%) | 0.88 |
| obesity, N=89 | 45 (6.5%) | 31 (6.1%) | 0.791 |
| heart failure, N=19 | 8 (1.1%) | 9 (1.8%) | 0.368 |
| nephrotic syndrome, N=2 | 1 (0.1%) | 1 (0.2%) | 0.824 |
| thrombophilia, N=37 | 15 (2.2%) | 22 (4.3%) | 0.031 |
| poly/thrombocytaemia, N=3 | 3 (0.4%) | 0 (0.0%) | 0.138 |
| antiphospholipid AB, N=5 | 4 (0.6%) | 1 (0.2%) | 0.313 |
| estrogens/progestins, N=61 | 15 (2.2%) | 44 (8.6%) | <0.001 |
| atrial fibrillation, N=10 | 4 (0.6%) | 5 (1.0%) | 0.417 |
| caval filter, N=5 | 1 (0.1%) | 4 (0.8%) | 0.087 |
| long travel, N=42 | 19 (2.7%) | 22 (4.3%) | 0.132 |

* the 35 cases of isolated PE were not classified

Supplemental table S-2. Distribution of risk factors for thrombosis by index event

| risk factor | proximal/proximal+distal DVT | isolated distal DVT | DVT+PE | PE without DVT | P (chi square) |
| --- | --- | --- | --- | --- | --- |
| surgery, N=198 | 85 (13.0%) | 94 (21.9%) | 16 (13.0%) | 3 (8.6%) | 0.001 |
| severe trauma, N=127 | 60 (9.2%) | 60 (14.0%) | 6 (4.9%) | 1 (2.9%) | 0.004 |
| cancer, N=85 | 38 (5.8%) | 38 (8.8%) | 7 (5.7%) | 2 (5.7%) | 0.256 |
| paraplegia, N=12 | 7 (1.1%) | 5 (1.2%) | 0 (0.0%) | 0 (0.0%) | 0.617 |
| limb immobilization, N=91 | 53 (8.1%) | 30 (7.0%) | 5 (4.1%) | 3 (8.6%) | 0.441 |
| central venous catheter, N=8 | 5 (0.8%) | 3 (0.7%) | 0 (0.0%) | 0 (0.0%) | 0.754 |
| chronic inflammatory disease, N=28 | 15 (2.3%) | 7 (1.6%) | 4 (3.3%) | 2 (5.7%) | 0.358 |
| bedrest >4 days, N=144 | 64 (9.8%) | 63 (14.7%) | 12 (9.8%) | 5 (14.3%) | 0.087 |
| pregnancy, N=21 | 16 (5.0%) | 3 (1.2%) | 2 (3.2%) | 0 (0.0%) | 0.073 |
| puerperium, N=8 | 5 (1.6%) | 2 (0.8%) | 0 (0.0%) | 1 (4.3%) | 0.359 |
| obesity, N=89 | 39 (6.0%) | 29 (6.7%) | 8 (6.5%) | 13 (37.1%) | <0.001 |
| heart failure, N=19 | 9 (1.4%) | 5 (1.2%) | 3 (2.4%) | 2 (5.7%) | 0.157 |
| nephrotic syndrome, N=2 | 1 (0.2%) | 1 (0.2%) | 0 (0.0%) | 0 (0.0%) | 0.942 |
| thrombophilia, N=37 | 18 (2.8%) | 11 (2.6%) | 8 (6.5%) | 0 (0.0%) | 0.081 |
| poly/thrombocytaemia, N=3 | 1 (0.2%) | 1 (0.2%) | 1 (0.8%) | 0 (0.0%) | 0.581 |
| antiphospholipid antibodies, N=5 | 2 (0.3%) | 1 (0.2%) | 2 (1.6%) | 0 (0.0%) | 0.159 |
| estrogens/progestins, N=61 | 27 (4.1%) | 20 (4.7%) | 12 (9.8%) | 2 (5.7%) | 0.068 |
| atrial fibrillation, N=10 | 2 (0.3%) | 5 (1.2%) | 2 (1.6%) | 1 (2.9%) | 0.133 |
| caval filter, N=5 | 2 (0.3%) | 3 (0.7%) | 0 (0.0%) | 0 (0.0%) | 0.633 |
| long travel, N=42 | 22 (3.4%) | 15 (3.5%) | 4 (3.3%) | 1 (2.9%) | 0.997 |

Supplemental Table S-3. Distribution of treatments and decision by index event

|  |  | proximal/ proximal+distal DVT | isolated distal DVT | DVT+PE | PE without DVT | P (chi square) |
| --- | --- | --- | --- | --- | --- | --- |
| treatment | VKA, N=159 | 92 (14.1%) | 47 (10.9%) | 11 (8.9%) | 9 (25.7%) | 0.027 |
|  | LMWH, N=80 | 35 (5.4%) | 34 (7.9%) | 11 (8.9%) | 0 (0.0%) | 0.091 |
|  | DOAC, N=975 | 513 (78.7%) | 341 (79.3%) | 98 (79.7%) | 23 (65.7%) | 0.299 |
|  | Other, N=33 | 15 (2.3%) | 12 (2.8%) | 3 (2.4%) | 3 (8.6%) | 0.165 |
| decision | continue with anticoagulation | 379 (58.1%) | 159 (37.0%) | 73 (59.3%) | 25 (71.4%) | <0.001 |
|  | continue with other antithrombotic | 187 (28.7%) | 123 (28.6%) | 30 (24.4%) | 9 (25.7%) | 0.778 |
|  | stop | 86 (13.2%) | 148 (34.4%) | 20 (16.3%) | 1 (2.9%) | <0.001 |

Supplemental Table S-4. Distribution of treatments and decision by type of index event

|  |  | unprovoked | provoked | P (chi square) |
| --- | --- | --- | --- | --- |
| event* | proximal/proximal+distal DVT, N=652 | 396 (56.9%) | 256 (50.3%) | 0.023 |
|  | isolated distal DVT, N=430 | 226 (32.5%) | 204 (40.1%) | 0.006 |
|  | DVT+PE, N=123 | 74 (10.6%) | 49 (9.6%) | 0.569 |
| treatment** | VKA, N=150 | 87 (12.5%) | 63 (12.4%) | 0.949 |
|  | LMWH, N=80 | 40 (5.7%) | 40 (7.9%) | 0.148 |
|  | DOAC, N=952 | 556 (79.9%) | 396 (77.8%) | 0.380 |
| decision | continue with anticoagulation, N=611 | 360 (51.7%) | 251 (49.3%) | 0.408 |
|  | continue with other antithrombotic, N=340 | 229 (32.9%) | 111 (21.8%) | <0.001 |
|  | stop, N=254 | 107 (15.4%) | 147 (28.9%) | <0.001 |

* the 35 cases of isolated PE were not classified

** 30 subjects received other treatments

Supplemental Table S-5. Distribution of decision, overall and by type of index event, according to the availability of recent ultrasound at the time of decision

|  | decision | recent ultrasound, N (%) | | P (chi square) |
| --- | --- | --- | --- | --- |
|  |  | not available | available |  |
| total | N | 350 | 890 |  |
|  | continue anticoagulation | 204 (58.3%) | 432 (48.5%) | 0.002 |
|  | switch to antithrombotics | 62 (17.7%) | 287 (32.2%) | <0.001 |
|  | stop treatment | 84 (24.0%) | 890 (19.2%) | 0.061 |
| unprovoked | N | 198 | 498 |  |
|  | continue anticoagulation | 127 (64.1%) | 233 (46.8%) | <0.001 |
|  | switch to antithrombotics | 37 (18.7%) | 192 (38.6%) | <0.001 |
|  | stop treatment | 34 (17.2%) | 73 (14.7%) | 0.407 |
| provoked | N | 120 | 389 |  |
|  | continue anticoagulation | 55 (45.8%) | 196 (50.4%) | 0.383 |
|  | switch to antithrombotics | 16 (13.3%) | 95 (24.4%) | 0.010 |
|  | stop treatment | 49 (40.8%) | 98 (25.2%) | 0.001 |

Supplemental Table S-6 – Outcome of the multivariable ordinal logistic regression analysis

**Logistic regression, all subjects**

| Decision | predictor | B* | Std. Error | P |
| --- | --- | --- | --- | --- |
| Continue with anticoagulation vs. stop | sex, women vs. men | -0.182 | 0.159 | 0.253 |
|  | age, <65 vs. 75+ | -0.192 | 0.270 | 0.478 |
|  | age, 65-74 vs. 75+ | -0.311 | 0.291 | 0.285 |
|  | DVT vs. PE | -0.996 | 0.272 | 0.000 |
|  | concomitant diseases, no vs. yes | -0.397 | 0.167 | 0.018 |
|  | risk factors, no vs. yes | 0.007 | 0.163 | 0.968 |
|  | China vs. Tunisia | 1.381 | 0.331 | 0.000 |
|  | Czech Rep. vs. Tunisia | -0.254 | 0.432 | 0.557 |
|  | Poland vs. Tunisia | 1.786 | 0.415 | 0.000 |
|  | Portugal vs. Tunisia | 1.451 | 0.480 | 0.003 |
|  | Russia vs. Tunisia | 1.511 | 0.325 | 0.000 |
|  | Slovakia vs. Tunisia | 2.970 | 0.674 | 0.000 |
| Continue with other antithrombotic vs. stop | sex, women vs. men | -0.120 | 0.178 | 0.500 |
|  | age, <65 vs. 75+ | 0.093 | 0.305 | 0.759 |
|  | age, 65-74 vs. 75+ | 0.124 | 0.327 | 0.705 |
|  | DVT vs. PE | -0.320 | 0.301 | 0.288 |
|  | concomitant diseases, no vs. yes | -0.561 | 0.189 | 0.003 |
|  | risk factors, no vs. yes | -0.124 | 0.182 | 0.496 |
|  | China vs. Tunisia | -0.444 | 0.327 | 0.174 |
|  | Czech Rep. vs. Tunisia | -0.369 | 0.392 | 0.346 |
|  | Poland vs. Tunisia | 1.032 | 0.399 | 0.010 |
|  | Portugal vs. Tunisia | -1.732 | 0.823 | 0.035 |
|  | Russia vs. Tunisia | 0.205 | 0.304 | 0.500 |
|  | Slovakia vs. Tunisia | 2.966 | 0.653 | 0.000 |

* the odds for a specific comparison with its CI is given by $e^{B\pm Z_{\alpha/2}\times SE}$; for the opposite comparison it is given by $e^{-B\pm Z_{\alpha/2}\times SE}$

**Logistic regression, only DVT subjects**

| Decision | Predictor | B* | Std. Error | Sig. |
| --- | --- | --- | --- | --- |
| Continue with anticoagulation vs. stop | sex, women vs. men | -0.103 | 0.166 | 0.537 |
|  | age, <65 vs. 75+ | 0.088 | 0.286 | 0.759 |
|  | age, 65-74 vs. 75+ | -0.159 | 0.306 | 0.604 |
|  | concomitant diseases, no vs. yes | -0.252 | 0.174 | 0.147 |
|  | risk factors, no vs. yes | 0.049 | 0.171 | 0.775 |
|  | DVT unprovoked vs. provoked | 0.630 | 0.162 | 0.000 |
|  | post-thrombotic syndrome, absent vs. present | -1.659 | 0.234 | 0.000 |
|  | China vs. Tunisia | 1.692 | 0.358 | 0.000 |
|  | Czech Rep. vs. Tunisia | -0.201 | 0.486 | 0.679 |
|  | Poland vs. Tunisia | 1.839 | 0.443 | 0.000 |
|  | Portugal vs. Tunisia | 1.504 | 0.507 | 0.003 |
|  | Russia vs. Tunisia | 1.241 | 0.351 | 0.000 |
|  | Slovakia vs. Tunisia | 3.196 | 0.687 | 0.000 |
| Continue with other antithrombotic vs. stop | sex, women vs. men | -0.062 | 0.184 | 0.737 |
|  | age, <65 vs. 75+ | 0.217 | 0.315 | 0.489 |
|  | age, 65-74 vs. 75+ | 0.086 | 0.337 | 0.798 |
|  | concomitant diseases, no vs. yes | -0.480 | 0.194 | 0.014 |
|  | risk factors, no vs. yes | -0.161 | 0.188 | 0.394 |
|  | DVT unprovoked vs. provoked | 0.962 | 0.183 | 0.000 |
|  | post-thrombotic syndrome, absent vs. present | -1.035 | 0.255 | 0.000 |
|  | China vs. Tunisia | -0.053 | 0.350 | 0.880 |
|  | Czech Rep. vs. Tunisia | 0.077 | 0.418 | 0.854 |
|  | Poland vs. Tunisia | 1.275 | 0.423 | 0.003 |
|  | Portugal vs. Tunisia | -1.531 | 0.837 | 0.067 |
|  | Russia vs. Tunisia | 0.219 | 0.329 | 0.506 |
|  | Slovakia vs. Tunisia | 3.259 | 0.666 | 0.000 |

* the odds for a specific comparison with its CI is given by $e^{B\pm Z_{\alpha/2}\times SE}$; for the opposite comparison it is given by $e^{-B\pm Z_{\alpha/2}\times SE}$

Supplemental Table S-7. Distribution of the reasons justifying the decision taken for continuation, stratified by decision taken

|  | continue with anticoagulation, (N=564*) | continue with other antithrombotic (N=329*) | stop (N=243*) | P (chi square) |
| --- | --- | --- | --- | --- |
| risk of recurrence | 481 (85.3%) | 251 (76.3%) | 101 (41.6%) | <0.001 |
| risk of bleeding | 222 (39.4%) | 160 (48.6%) | 91 (37.4%) | 0.008 |
| no treatment needed | 21 (3.7%) | 63 (19.1%) | 183 (75.3%) | <0.001 |
| patient's choice | 106 (18.8%) | 64 (19.5%) | 94 (38.7%) | <0.001 |
| D-dimer negative | 74 (13.1%) | 66 (20.1%) | 89 (36.6%) | <0.001 |
| PTS present | 138 (24.5%) | 53 (16.1%) | 7 (2.9%) | <0.001 |
| patient's age | 59 (10.5%) | 22 (6.7%) | 16 (6.6%) | 0.071 |
| cost for the patient | 19 (3.4%) | 9 (2.7%) | 43 (17.7%) | <0.001 |
| D-dimer positive | 45 (8.0%) | 12 (3.6%) | 6 (2.5%) | 0.001 |
| thrombophilia | 43 (7.6%) | 10 (3.0%) | 3 (1.2%) | <0.001 |
| active cancer | 45 (8.0%) | 5 (1.5%) | 1 (0.4%) | <0.001 |
| decision by GP | 21 (3.7%) | 10 (3.0%) | 10 (4.1%) | 0.776 |
| familiarity for VTE | 20 (3.5%) | 10 (3.0%) | 2 (0.8%) | 0.096 |
| implantation of caval filter | 27 (4.8%) | 2 (0.6%) | 2 (0.8%) | <0.001 |
| major bleeding on treatment | 4 (0.7%) | 15 (4.6%) | 2 (0.8%) | <0.001 |
| unstable INR level | 11 (2.0%) | 8 (2.4%) | 2 (0.8%) | 0.358 |
| tendency to fall | 8 (1.4%) | 4 (1.2%) | 8 (3.3%) | 0.12 |
| pulmonary hypertension | 11 (2.0%) | 4 (1.2%) | 0 (0.0%) | 0.082 |
| general conditions worsened | 11 (2.0%) | 1 (0.3%) | 0 (0.0%) | 0.013 |
| onset of contraindications | 2 (0.4%) | 1 (0.3%) | 2 (0.8%) | 0.593 |
| renal insufficiency worsened | 0 (0.0%) | 0 (0.0%) | 2 (0.8%) | 0.025 |

* N denotes the number of reasons given, not the number of patients
